# A Case of Doublets—Uterine Inertia—Alarming Sequences

**Published:** 1867-11

**Authors:** E. L. Connally

**Affiliations:** Albany Ga.


### ARTICLE IV.

### *A Case of Doublets—Uterine Inertia—Alarming Sequences.*

By E. L. Connally, M. D., Albany Ga.

A few months since I was called to Mrs.—— H., aged 28 years—had been married 14 months—nervous temperament —delicate, but apparently healthy. Found her seven or eight months advanced in first pregnancy. Patient complained of stranguary, with pain in the illiac region; and a sense of great weight and fullness about the pubis, which I found to arise from a vascular tumor of the meatus urinarius. It seemed probable that the illiac distress arose from tension of the uterine ligaments. As objection was made to the simple operation proposed to relieve the vascular tumor, and no other interference seemed necessary, a prescription incorporating Bromide of Potassa was made, at the same time expressing the hope that all would go well with her.—Before leaving, however, she informed me that previously to her marriage she had been treated for uterine disease by cauteration and pessaries.

On the 25th of May, at 8 a. m., was called in haste, and found her in the first stage of labor. Was unable to discover any dilatation of the os, and after giving some instructions as to the management of the case in my absence, left her for three hours. On my return found labor progressing slowly; os slightly dilated, soft and dilatable, but pains not very distinct, apparently. Patient complained of great suffering, general uneasiness, and nervous irritability. Dr. Cromwell, who accompanied me in this visit, suggested the inhalation of Chloroform, which was used to incomplete anæsthesia, quieting her into a comfortable slumber, in which consciousness was not entirely destroyed, but ease afforded. Continuing the anæsthetic, at short intervals and in small quantities, for several hours, it was found that the os had not dilated further during the anæsthesia, nor were there any other evidences of uterine contraction than agonizing pain in the back and pubic region.

The administration of ergot was then commenced in the dose of a teaspoonful of wine every thirty minutes.—Finding no impression made, after using it for an hour, the dose was increased to double that quantity, until two ounces had been consumed, with the same result. Seeing no good reason why the amount taken, if an active preparation, should not produce contraction of the womb, it was determined to try another specimen, which was done to extent, without any impression upon the uterus. Not being yet satisfied to give up my confidence in the efficiency of this useful drug, I obtained the same preparation of ergot from the third druggist. In the mean time a cup of coffee and an occasional drink of water had been taken, and had been ejected from the stomach before the administration of the third specimen of the ergot. The last two ounces were then given in the dose of a tablespoonful every twenty minutes; to which was, once or twice, added a drachm of Dr. Squibb's ext. ergot. She had then taken, during six hours, about six fluid ounces wine of ergot, and one or two drachms Squibb's extract, without having produced any perceptible impression, except occasional vomiting, during the administration.

It being then eighteen hours since the commencement of labor, and the patient expressing a sense of great exhaustion, at midnight an anodyne of opium was given and quiet enjoyed till morning. Being in the habitual use of Opium, and the stomach at the time irritable, half drachm Squibb's Comp. Liq. Opium was introduced under the skin by the hypodermic syringe. This gave quiet and comfort, to some extent, but did not induce sleep.

26th.—7, a. m.—Patient in pretty much same condition: os uteri soft and yielding, but very little more dilated than on the previous evening. After being propt up in bed, nourishment was ordered and I left her for two hours. Returned at ten o'clock, accompanied by my friend and partner Dr. Cromwell, and found her quiet, and the os considerably dilated—larger than a dollar; the child was lower in the pelvis, seemingly from the force of gravitation, as there were no pains by which to account for it or the dilation of the os, unless insensible contraction of the longitudinal fibres not discoverable to patient or physician was operating on the womb. All that seemed necessary to complete the delivery was two or three expulsive throes.

With the hope that the few hours rest had changed the susceptibility of the uterus, we again commenced the use of Ergot in full doses. The result was, however, as before—no contraction produced. We then determined to deliver with the forceps. She had been in labor thirty hours. While under the influence of Chloroform the streight forceps were applied and she delivered of an asphyxed female child. It was, however, soon resuscitated by Dr. C., and in the mean time I found descending the head of an other child. The forceps were again applied, and she was delivered of another female child. This was also with difficulty resuscitated. The secundines were immediately remomved, but the uterus did not contract promptly. Considerable hemorrhage supervened, and continued till the hand containing a lump of ice of considerable size was introduced into the womb and rotated so as to excite the organ to the necessary contraction.

The children, after new life was established, were apparently healthy, weighing six pounds each. Prescribed for the mother Brandy and Morphine.

9 p. m.—Patient comfortable, but had not slept. Some uneasiness from distension of the bladder, which was relieved by the Catheter. Ordered half grain Morphine, and repeated in two hours if sleep was not induced.

May 27, 9 a. m.—No sleep during the night. Relieved the bladder of a large quantity of urine. Symtoms favorable, except that she had not slept since the commencement of labor, and particularly loquacious. There was evidently a tendency to mental aberation, of which she had, to some extent, suffered before her marriage. In order to quiet the mental agitation and promote sleep, I gave hypodermically eight drops solution of Morphia and Atropia, containing one-fourth grain of the former and one eightieth grain of the latter. Not finding any impression made in twenty minutes, twelve drops of the same solution were injected under the skin. After waiting thirty minutes, without any decided impression, and knowing that the habitual use of Opium before confinement made it necessary to use more than would otherwise be necessary, the third dose containing twelve drops of the solution was administered hypodermically. She had then taken, in the course of an hour, one grain of Morphine and one-twentieth grain Atropia. After remaining half an hour and finding no disposition to sleep, but still very talkative, I enjoined perfect quiet in the room and left.

In about three hours was summoned in haste, and found her insensible; pulse labored, strong and frequent; breathing difficult. Supposing the symptoms were probably the result of narcotism, I ordered coffee, and as soon as it could be obtained, injected caffien under the skin. In the mean time, however, the symptoms of asphyxia increased, until the face became livid, and the breathing for a time entirely interrupted. Finally the breathing became easier under the influence of caffeine, cold applications to the head, sinapisms to the spine and extremities. For an hour or two she thus remained, breathing imperfectly—pulse 140 and full, when suddenly she sprang in bed to a sitting posture, with unintelligible and incoherent expressions. Soon she became composed, and talked to some extent rationally, of her having realized the torments of hell! The peculiar maniacal stare of the eyes soon gave place to a more tranquil expression, and with it her accustomed loquacity. A blister was applied to the abdomen and the bladder emptied.

Returned at midnight, and though more composed, in every way, found her still talking.

May 28, 8 a. m.—Has slept very little during the night—not half an hour, all together. Pulse 100—still talking. Took nourishment and nursed her children, of whom she seemed very fond. Prescribed Ol Ricini, and evacuated bladder.

2 p. m.—Bowels acting freely from oil. Prescribed a drachm Tr. Opium by enema.

9 p. m.—Had not slept; continued to talk incessantly; pulse 105. Emptied the bladder, and prescribed, hypodermically, three-fourths grain Morphia and one-fiftieth grain Atropia.

May 30, 9 a. m.—Has slept but few moments during the night—Catheter used at midnight. Pulse 100; tongue coated with a heavy, buffy fur. Prescribed the following:

℞ Hydrarg. Chlor. Mite grs. v.
Pulv. Rhei grs. x.
M.

S.—Take at once in electuary.

Catheterism, and the occasional use of some opiate preparation constituted the principal treatment for several days.

June 4, 9 a. m.—Symptoms not so favorable. Pulse 130 and hard; tongue red, smooth, dry and pointed; pain in the head; lactation considerable, with pain and soreness of the mammæ—no tenderness of the abdomen. Prescribed the following:

℞ Hydrarg. Chlor. Mite grs. vj.
Pulv. Doveri ℈ ji.
M. and make six papers.
S.—One every three hours.

In addition, iced acacia water for a constant drink, the breast pump and Ext. Belladonna to the Mammæ, were directed.

10 p. m.—Free perspiration; pulse 115 and soft.

June 5, 9 a. m.—Pulse 98; had slept about two hours during the night. Prescribed the following:

℞ Quinia Sulph. grs. xxji.

Acid Sulph. Asom. grs. [illegible]

M. and Ft. Pil. No. xvj.

S.—Two every three hours.

June 10, 7 a. m.—Complained of uneasiness and pain in the bladder; examined the urine and found considerable pus discharged with it. Injected into the bladder tepid water, and after washing out the viscus, threw into it tepid water acidulated with Acidum Nitrici—afterwards a solution of Hyoscyamus. For internal administration prescribed emulsion of Terpentine, Quinine and Iron, nourishing diet and brandy.

June 23.—Patient feeble; prognosis more unfavorable—growing weaker, despite the supporting treatment. The condition of the bladder, however, very much improved.—Suffers pain in the right lumbar region and centre of the sternim, without any perceptible cause. Persistent insomnia and retention of the urine are the constant difficulties through the whole progress of the case, with unabating loquacity. Great emaciation had resulted from the protracted suffering. Prescribed the following:

℞ Ext. Hyoscyami grs. xx.
Ext. Strammonii grs. jii.
Morphæ Sulph. grs. ii.
M. and Ft. Pil. No. vj.
S.—One every three hours.

Under this preparation she rested more quietly, but slept very little. From the soothing influence of these neurotics her strength somewhat increased. For a few days after the use of the above combination all medicine was suspended; then the same repeated. In the mean time the left breast had suppurated and discharged a large amount of pus. The issue continued for about six weeks.

July 1.—Discharge from the breast very much lessened; voids urine. Under the use of Aromatic Sulph. Acid and Quinine the appetite has very much inproved, and the general strength greatly increased; sleeps comparatively well.

The twins died when three weeks old from tabes mesenterica.

The principal points of interest to me in this case are:

1st. The cessation of uterine contractions during partial anæsthesia from Chloroform.

2d. The failure to reëstablish uterine action by the use of Ergot.

3d. Was the failure dependent on the distention of the organ, from the anæsthesia, or from opiates given during the labor?

4th. The protracted insomnia and incessant volubility, which resisted every means resorted to to overcome it.

